# Ultrasound-Guided Femoral and Sciatic Nerve Blocks for Repair of Tibia and Fibula Fractures in a Bennett's Wallaby (*Macropus rufogriseus*)

**DOI:** 10.1155/2016/8909205

**Published:** 2016-10-10

**Authors:** Paolo Monticelli, Luis Campoy, Chiara Adami

**Affiliations:** ^1^Department of Clinical Sciences and Services, Royal Veterinary College, University of London, Hatfield, Hertfordshire AL97TA, UK; ^2^Department of Anesthesiology and Analgesia, College of Veterinary Medicine, Cornell University, Ithaca, NY 14853, USA

## Abstract

Locoregional anesthetic techniques may be a very useful tool for the anesthetic management of wallabies with injuries of the pelvic limbs and may help to prevent capture myopathies resulting from stress and systemic opioids' administration. This report describes the use of ultrasound-guided femoral and sciatic nerve blocks in Bennett's wallaby (*Macropus rufogriseus*) referred for orthopaedic surgery. Ultrasound-guided femoral and sciatic nerve blocks were attempted at the femoral triangle and proximal thigh level, respectively. Whilst the sciatic nerve could be easily visualised, the femoral nerve could not be readily identified. Only the sciatic nerve was therefore blocked with ropivacaine, and methadone was administered as rescue analgesic. The ultrasound images were stored and sent for external review. Anesthesia and recovery were uneventful and the wallaby was discharged two days postoperatively. At the time of writing, it is challenging to provide safe and effective analgesia to Macropods. Detailed knowledge of the anatomy of these species is at the basis of successful locoregional anesthesia. The development of novel analgesic techniques suitable for wallabies would represent an important step forward in this field and help the clinicians dealing with these species to improve their perianesthetic management.

## 1. Introduction

In this report we describe the use of ultrasound-guided femoral and sciatic nerve blocks in a wallaby undergoing surgical fixation of tibial and fibular fractures. This technique [1] is commonly used in our referral hospital for patients undergoing orthopaedic surgeries involving the stifle joint or structures located distal to it. Its analgesic efficacy has been demonstrated in various animal species, including dogs, goats, pet rabbits, and raptors [2–5].

It is reasonable to assume that, similarly to other animal species, also wallabies would benefit from femoral-sciatic block as a part of the management of the pelvic limb surgeries. Furthermore, Macropods have unique physiological features which make locoregional anesthesia a particularly attractive option for the treatment of perioperative pain. As wild animal species, they are prone to develop a life-threatening condition known as capture myopathy, of which opioids administration and stressors, such as physical restraint and poorly managed pain, are well-recognised triggering factors [6, 7]. Within the last decades, a lot of research has been done with the purpose of developing novel techniques to minimise the stress response and its fatal sequels [8, 9], and it has been demonstrated that the choice of the anesthetic technique does play a major role in decreasing the incidence of these events [8]. As a matter of fact, successful locoregional anesthesia is likely to have a sparing effect on intraoperative requirement of opioids, which are believed to be implicated in the pathogenesis of rhabdomyolysis possibly through vasospasm, hyperpyrexia, and direct myofibrillar damage [6]. Similarly, poorly managed pain and the resulting stress would cause decreased muscular perfusion, lactic acidosis, and adenosine triphosphate depletion through a prolonged adrenergic stimulation [6, 7, 9].

When selecting a locoregional anesthetic technique for wallabies, some species-specific consideration should be taken into account. One advantage of peripheral nerve blocks over neuroaxial anesthesia is the unilateral versus bilateral motor blockade, an aspect particularly desirable in wild Macropods, whose early regain of pelvic limbs' motor function is of primary importance to minimise the stress response and to increase the chance of postsurgical survival. In the light of these considerations, sciatic-femoral block was regarded by the authors as a suitable option to provide analgesia to the wallaby object of this report, undergoing surgical treatment of comminuted tibial and fibular fractures.

The aim of this report was to describe the technical aspects of this locoregional technique and to discuss the encountered challenges, when applied to wallabies.

## 2. Case Presentation

Two-month-old female entire pet Bennett's wallaby (*Macropus rufogriseus*) weighing 4 Kg was referred to the Queen Mother Hospital for Animals (QMHA) for diagnosis and treatment of sudden onset-left pelvic limb lameness. A protective bandage had been applied by the referral veterinarian and buprenorphine (20 mcg/kg) was administered intramuscularly (IM) every 8 hours. The wallaby was scheduled for diagnostic imaging and possibly surgery on the following day.

The wallaby was ambulatory on presentation and appeared bright, alert, and responsive. Resting heart rate was 144 beats/min with regular rhythm. Thoracic auscultation was unremarkable with a respiratory rate of 28 breaths/min. Preanesthetic blood work results were not available. The radiographic exam, performed in the anaesthetised animal, showed a complete, mild comminuted fracture of the distal physis of the tibia affecting the distal part of the adjacent metaphysis. The fracture was slightly displaced craniolaterally. A complete, short oblique fracture of the distal third of the fibula was also visible. Moderate swelling extending along the crus was also present. It was decided to proceed with surgical fracture fixation and cast application.

Food and drinking water were withdrawn immediately before anesthesia. The wallaby was tail restrained [10] to allow injection of 50 mcg/kg of dexmedetomidine and 5 mg/kg of ketamine in the left quadriceps. Onset of anesthesia was approximately 5 minutes from injection. Thereafter, a 22-gauge catheter was inserted in the right cephalic vein and the wallaby was positioned in dorsal recumbency with the neck extended to allow endotracheal (ET) intubation. The latter was accomplished with a 3.0 mm ID cuffed ET-tube, inserted orotracheally under direct laryngoscopy through an “over the catheter” technique. The ET-tube was then connected to modified Ayre's T-piece and the fresh gas flow rate was adjusted in order to prevent carbon dioxide rebreathing. The inspired fraction of oxygen (F_i_O_2_) was 1. Anesthesia was maintained with sevoflurane vaporised in oxygen (end-tidal: 1.5–2.0%). Crystalloids (Lactated Ringer's) were infused intravenously (IV) at a rate of 5 mL/kg/h. A multiparametric module (S/5 Datex-Ohmeda) was used to continuously monitor physiological parameters, which were manually recorded at a 5-minute interval. The systolic, mean, and diastolic arterial blood pressure (SAP, MAP, and DAP, resp.) were measured noninvasively (oscillometry technique), with a size 4-cuff placed at the base of the tail over the coccygeal artery. During the anesthetic, the wallaby was allowed to breathe spontaneously with a respiratory rate ranging from 20 to 30 breaths/min. No further rescue analgesics were required during surgery. Intraoperatively, moderate bradycardia (65 beats/min) and associated hypotension (MAP 45 mmHg) occurred approximately 45 minutes after anesthetic induction and were initially treated with IV glycopyrronium (10 mcg/kg). Due to the lack of response in heart rate and persistent hypotension, a dopamine infusion was commenced 10 minutes later at a rate of 5 mcg/kg/min. Subsequently, heart rate returned to baseline values and MAP increased to 75 mmHg and both variables were maintained at physiological values until the end of the procedure.

In order to provide perioperative analgesia, an ultrasound- (US-) guided combined femoral and sciatic nerve block was attempted as previously described by Campoy and colleagues in the dog [1]. An ultrasonographer (S9v, Sonoscape, China) equipped with a 25 mm linear-array transducer (10–6 MHz) was used.

**Figure 1:**
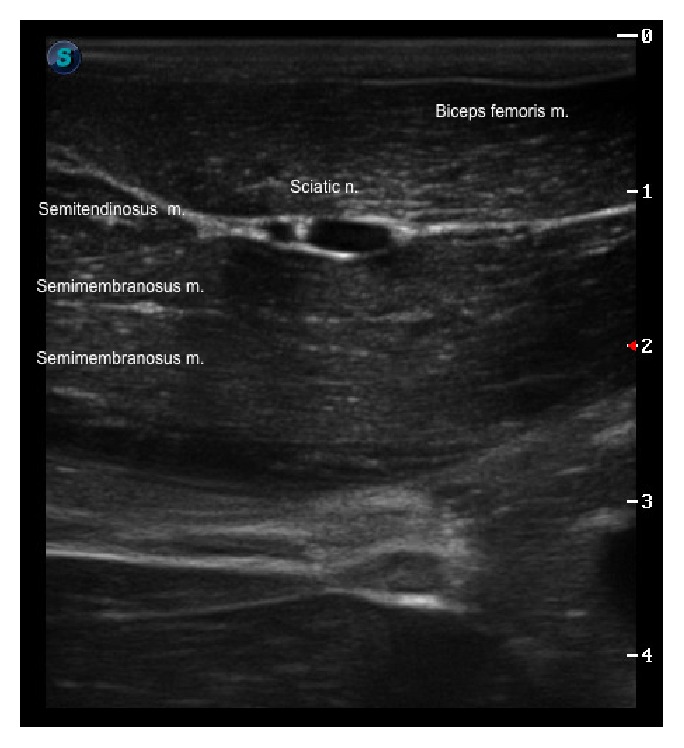
Sciatic nerve: ultrasonographic short axis view image of the sciatic nerve of a wallaby (*Macropus rufogriseus*). The sciatic nerve can be identified as a hypoechoic double-ellipsoid structure located deep to the fascia of the* biceps femoris* muscle and cranial and superficial to the two bellies of the semimembranosus muscle, respectively. S is caudal.

Briefly, the sciatic nerve block was performed with the wallaby in lateral recumbency and the affected limb positioned uppermost. The ischiatic tuberosity and the greater trochanter were identified by palpation and used as anatomical landmarks. The transducer was then positioned immediately distal to these landmarks, on a transverse plane with respect to the long axis of the femur in an attempt to obtain a short axis view of the sciatic nerve. The sciatic nerve was identified as a hypoechoic rounded structure (Figure 1) located between the hyperechoic fascias of the* biceps femoris*, the abductor, and the two bellies of the semimembranosus muscles. Subsequently, a 22 G, 63 mm Quincke spinal needle was advanced “in-plane” from the caudal aspect of the thigh towards the nerve. Once the needle could be visualised in the proximity to the sciatic nerve, an aspiration test was performed to rule out intravascular location of the tip of the needle. Ropivacaine 0.75% was injected at a volume of 0.05 mL/kg. The correct perineural position of the needle was confirmed by the observation of even spread of the local anesthetic around the sciatic nerve.

The ultrasonographic identification of the femoral nerve was more challenging. As a consequence, additional nerve stimulator guidance was used.

**Figure 2:**
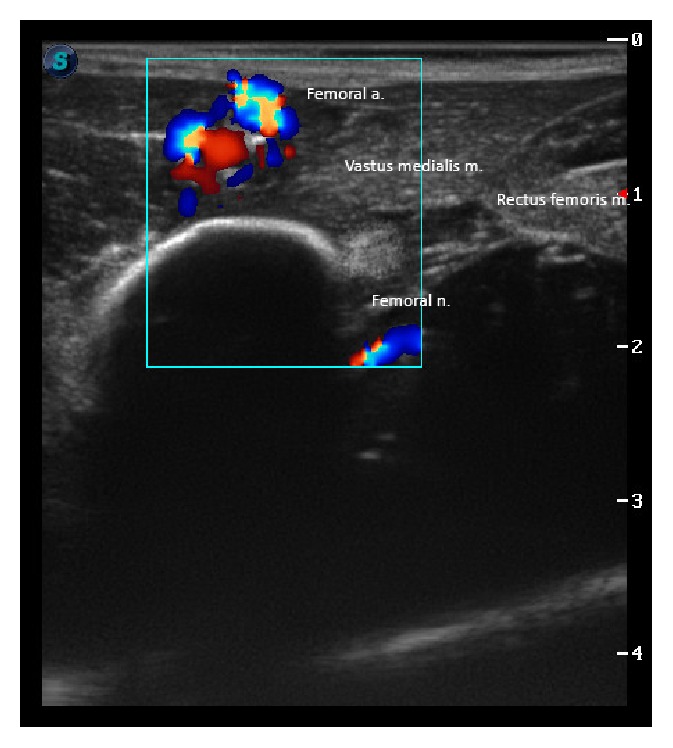
Femoral nerve: ultrasonographic view of the inguinal area of a wallaby (*Macropus rufogriseus*). The transducer was positioned on a transverse plane with respect to the long axis of the femur to obtain a short axis view of the femoral nerve. The femoral nerve can be identified as a hyperechoic rounded structure cranial to the femur, caudal to the* rectus femoris* muscle, and deep to the femoral artery and* vastus medialis* muscle. S is caudal.

Briefly, with the animal in right lateral recumbency, the left pelvic limb was abducted 90° and slightly extended caudally, and the transducer was positioned on a transverse plane with respect to the long axis of the femur in order to obtain a short axis view of the femoral nerve. The femoral artery was located by pulse palpation within the femoral triangle. The femoral nerve, as previously described in the dog [1], should be typically identified as a hyperechoic nodular structure located cranial and deep to the femoral artery and the fascia iliaca and caudal to the* rectus femoris* muscle. However, this did not seem to be the case and the targeted nerve could not be clearly identified on an initial attempt. A transient, weak motor response, characterised by extension of the knee and contraction of the quadriceps muscle, could only be evoked at currents greater than 1 mA. The block was aborted and 0.1 mg/kg of methadone was administered IV. Before transferring the patient to the surgery theatre, the ultrasound images of the femoral triangle were stored and sent off seeking a second opinion (Figure 2).

The fracture was reduced via a combined medial and lateral approach. A 0.8 mm K wire was placed as an intramedullary pin within the fibula maintaining limb alignment. Following fibular intramedullary pinning, a 1.2 mm K wire was driven from the medial malleolus across the fracture line to engage the transcortex of the tibia.

General anesthesia lasted 4 hours. Postoperative radiographs demonstrated appropriate fracture reduction and implant positioning. After a cast was applied to the operated limb, the wallaby was transferred to recovery for the early postoperative care.

Despite the use of both active and passive warming devices (two Bair Huggers plus an isolating blanket between the patient and the operating table) throughout anesthesia, moderate hypothermia (rectal temperature: 32.4°C) was observed [9, 11]. Active warming was continued during the early recovery period and the wallaby started eating grass as soon as its rectal body temperature reached 36.5°C, which happened one hour after tracheal extubation. Buprenorphine, 20 mcg/kg, was administered IV 4 hours after the administration of intraoperative methadone. No motor block was observed in the immediate postoperative period.

Physical and radiographic examination performed 6 weeks after surgery revealed a good clinical condition of the wallaby, with normal orthopaedic and neurological function of the operated limb.

## 3. Discussion

This report describes the challenges encountered when applying the technique described to perform femoral-sciatic nerve block in dogs to a wallaby, undergoing hind limb orthopaedic surgery.

Whilst ultrasonographic identification of the sciatic nerve could be achieved easily, although both operators were familiar with the use of these locoregional techniques in dogs, blocking the femoral nerve in this wallaby was more challenging than expected. A careful, more attentive analysis of the stored ultrasound images carried out at a later stage revealed the slightly different location of the femoral nerve compared with that in the dog. It is difficult to formulate a convincing hypothesis to explain this finding. One possibility may be that the unique hopping locomotion of marsupials implies peculiar geometry of the hind legs, which is accomplished by a different spatial and anatomical arrangement of its related muscles, tendons, and nerves compared to quadrupeds. During hopping, the muscles that deliver the main driving force and those involved in retraction are grouped around the hip, whereas the muscles of the lower, more distal areas store the elastic energy during landing at the end of a hop [12, 13]. Additionally, a marsupial lands all the large muscles of the hind legs which are in an active state simultaneously, unlike in quadrupeds in which the muscles in one leg are relaxed whilst those in the other leg are contracting [12, 13]. These unique anatomical, geometrical, and physiological features of the pelvic limbs of wallabies may explain the slightly different spatial arrangement of the femoral nerve compared to dogs. Nevertheless, it is worth emphasising that the same anatomical features were still preserved. The location of the femoral nerve was still deep to the sartorius muscle, femoral artery, and* vastus medialis* muscle and caudal to the* rectus femoris*. The fact that the* vastus medialis* could be identified on the ultrasound images may indicate a slightly distal location of the transducer compared to the classical approach as described in dogs or alternatively an overdeveloped* vastus medialis* extending more proximally than in the dog. The former theory may agree with the fact that when the targeted structure was electrically stimulated, only currents greater than 1 mA could elicit a mild response of the quadriceps. This seems to indicate that the nerve identified at this location was the saphenous rather than the femoral nerve. In retrospect, the administration of local anesthetic around any of these nerves would have likely yielded a successful blockade.

With respect to the sciatic nerve block performed in the wallaby, the administration of preoperative methadone makes any consideration regarding the analgesic efficacy of the block a speculation. To the best of the authors' knowledge, there are no published data about the pharmacological properties of methadone in* Macropus rufogriseus*. Nevertheless, methadone was administered to the wallaby only once and at a dose lower than the one generally recommended for dogs. Based on our clinical experience, it is unlikely that methadone, used as sole anesthetic at such low dose, would result in satisfactory analgesia in dogs undergoing invasive orthopaedic procedures. Furthermore, the wallaby had a comminuted fracture whose surgical fixation required considerable manipulation of bones and soft tissues, which presumably resulted in intense nociceptive stimulation. This seems to suggest that, to some extent, the sciatic nerve block contributed to providing analgesia to the wallaby during surgery. However, the paucity of scientific literature focusing on analgesia in Macropods, together with the lack of validated scales for recognition of pain in these species, makes proper assessment and treatment of pain a goal very difficult to achieve.

Also the pharmacokinetics and the pharmacodynamics of local anesthetic agents are largely unknown in marsupials. In the wallaby object of this report, it was decided to use ropivacaine for the sciatic nerve block, owing to its favourable pharmacological profile in both humans and dogs, and no adverse effects were observed. Ropivacaine is a long-lasting amino amide whose efficacy is comparable to the one of bupivacaine but with a lower propensity to produce motor block [14] and cardiovascular or neurological toxicity [15]. These features make ropivacaine a more attractive option than bupivacaine in animal species in which there is paucity of literature and an early regain of motor function of the hind limbs is of crucial importance.

In conclusion, with some limitations, this case report highlights the potential usefulness of locoregional anesthetic techniques in wallabies undergoing pelvic limb surgery. The peculiar anatomy of the nerves and muscles of the hind limbs of marsupials should be taken into account when attempting sciatic and femoral nerve blocks. The ultrasonographic study of the area may represent a first step for the development of species-specific locoregional techniques to be used in wallabies.
